# A Minimally Invasive Approach for Immediate Palatal Implant Placement with Osseodensification and Dynamic Navigation: Two Case Reports

**DOI:** 10.7759/cureus.102097

**Published:** 2026-01-22

**Authors:** Franco Ignáccio Mallaguti, José Geraldo Malaguti, Cássio C Orth

**Affiliations:** 1 Implantology, Malaguti Institute of Advanced Dental Technologies, Uberaba, BRA; 2 Periodontics, ImplantePerio, São Paulo, BRA

**Keywords:** case report, dental, dental implants, dynamic navigation, esthetics, humans, osseodensification, root resorption

## Abstract

Implant placement in the posterior maxilla is challenging due to low bone density and sinus pneumatization, which limits bone height. Indirect sinus elevation with osseodensification (OD) can compact trabecular bone while preserving sinus integrity and shortening overall treatment time. Dynamic navigation (DN) enhances real-time accuracy and predictability, and palatal positioning may help preserve the three-dimensional soft-tissue architecture.Two patients underwent immediate implant placement in the posterior palatal bone following extraction combined with indirect sinus floor elevation using OD burs and DN. Immediate provisionalization was performed in both cases to support soft tissue architecture, yielding predictability with reduced morbidity and overall treatment time.The combination of palatal implant placement, indirect sinus elevation by OD, and DN followed by immediate provisionalization offers a minimally invasive, predictable, and time-efficient strategy for rehabilitating challenging posterior maxillary sites. Although promising, validation through larger studies with longer follow-up is needed.

## Introduction

Oral rehabilitation with osseointegrated implants has become a well-established practice in contemporary dentistry, providing predictable functional and esthetic outcomes in various clinical scenarios [1]. However, implant placement in the posterior maxilla remains a technical challenge owing to low bone density and maxillary sinus pneumatization, which may limit the available vertical bone height [1,2].

Maxillary sinus elevation techniques, either direct or indirect, have been widely used to enable implant placement. Among the indirect approaches, the use of osseodensification (OD) has gained prominence due to its ability to preserve sinus integrity while simultaneously enhancing the osteotomy site quality through trabecular bone compaction and redirection. Unlike conventional techniques, OD comprises the use of specially designed drills to densify and compact bone during osteotomy preparation, optimizing regeneration and increasing bone density, thereby enhancing primary and secondary implant stability. OD is an additive osteotomy technique that does not involve excavation. When OD burs are operated in a noncutting direction (counterclockwise) with sufficient irrigation, they generate a hydraulic wave at the point of contact, compacting and autografting bone into the trabecular space both apically and laterally. Moreover, this technique has been associated with reduced overall treatment time [1-3].

Additionally, the evolution of digital technology in implant dentistry has significantly improved surgical precision. Among the available tools, dynamic navigation (DN) surgery systems enable real-time monitoring of the drill position and angulation during osteotomy and implant placement, thereby increasing predictability and reducing surgical risks. DN in implant dentistry uses systems that integrate cone-beam computed tomography (CBCT) with intraoral fiducial registration, virtual implant planning software, and a streamlined clinical setup, enabling an efficient workflow that can be applied routinely to virtually every patient receiving a dental implant. A key advantage is the ability to make intraoperative adjustments when needed, including changes in implant size, length, diameter, shape, and even positioning, according to clinical requirements to achieve the most accurate final placement. In essence, DN provides real-time coordination between the surgeon’s hands and eyes through highly magnified 3-dimensional (3D) visualization of the osteotomy preparation, making it essential that accuracy and precision are properly established and validated for any navigation system used [4,5].

Implant placement in the palatal bone of the posterior maxilla may be a viable alternative in cases of extensive periodontal and/or periapical lesions associated with maxillary sinus pneumatization [1,2]. When combined with indirect sinus floor elevation, this approach may reduce surgical morbidity and overall treatment time [3]. Immediate palatal implant placement may also offer advantages such as preservation of the buccal bone plate and peri-implant soft-tissue contours, improved restorative feasibility in thin ridges by avoiding buccal fenestration/dehiscence, and in selected cases, a reduced need for additional grafting with greater soft-tissue stability. However, without prosthetically driven planning, a palatal trajectory can introduce restorative challenges, including an unfavorable screw-access emergence, the need for angled components or customized abutments, and buccal overcontouring to re-establish the arch form, which may compromise cleanability, increase food impaction, and adversely affect the emergence profile. Moreover, limited access and the requirement for precise 3D orientation in the posterior maxilla further underscore the value of adjunctive tools such as DN systems [4,5].

This study aimed to report two clinical cases in which implants were placed in the posterior palatal bone immediately following tooth extraction, combined with indirect sinus elevation using OD burs and the DN system. In both cases, immediate provisionalization was performed to preserve tissue architecture and optimize both functional and esthetic outcomes. This report is novel in that it integrates immediate palatal implant placement with OD mediated indirect sinus floor elevation and DN in a single workflow, aiming to enhance accuracy, primary stability, and restorative predictability in the posterior maxilla.

## Case presentation

Case 1

**Figure 1:**
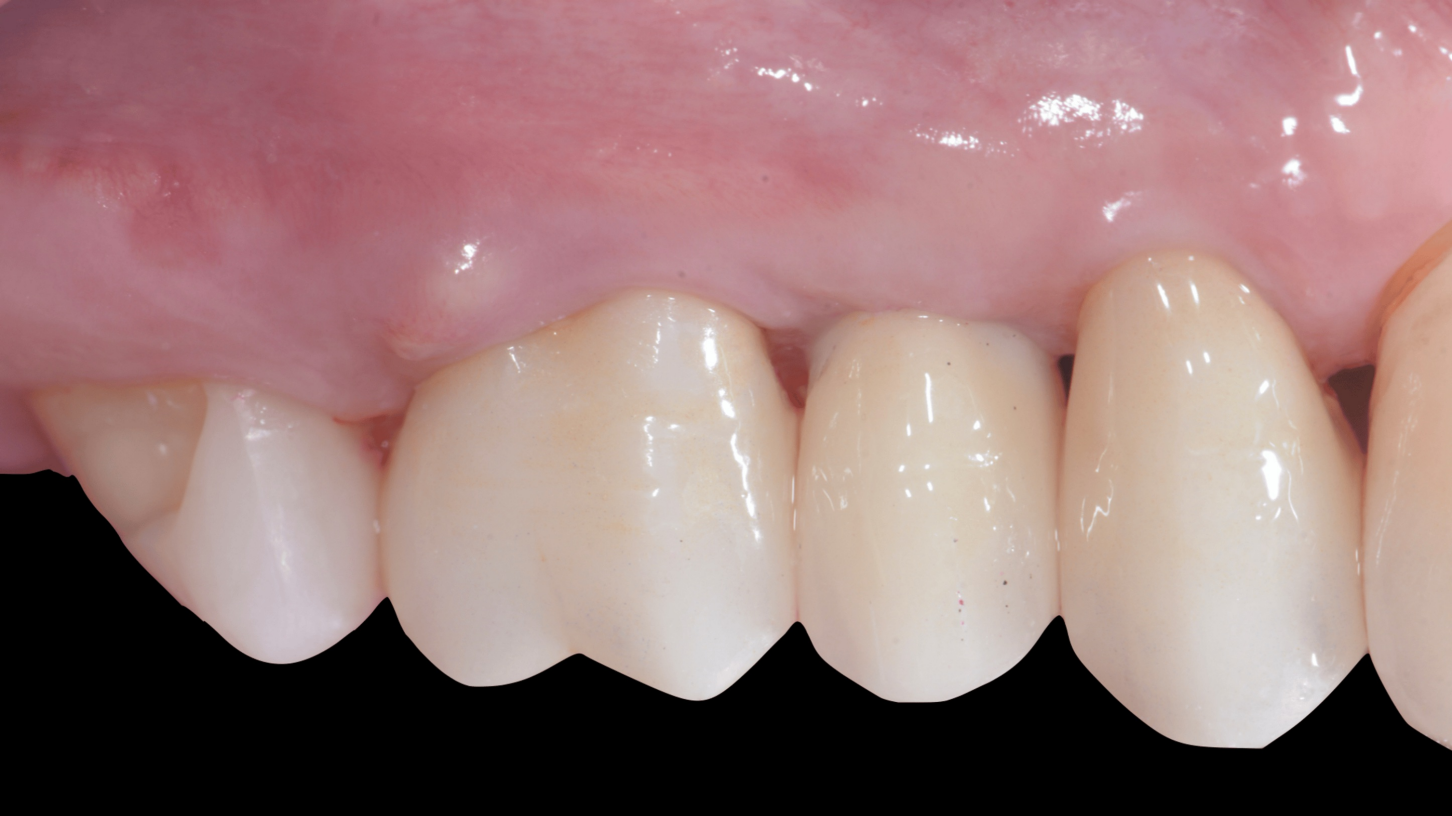
Clinical photograph of the area of chief complaint showing a fistula in the keratinized mucosa at tooth 16 and a pre-existing complete crown fracture at tooth 17

A 77-year-old male patient, M.T.P., was referred with a chief complaint of discomfort in quadrant 1, particularly in the region of tooth 16, and a crown fracture of tooth 17. Clinical examination revealed a fistula at the buccal aspect of the first molar (Figure 1) with a 7 mm probing depth and tenderness to vertical percussion. 

**Figure 2:**
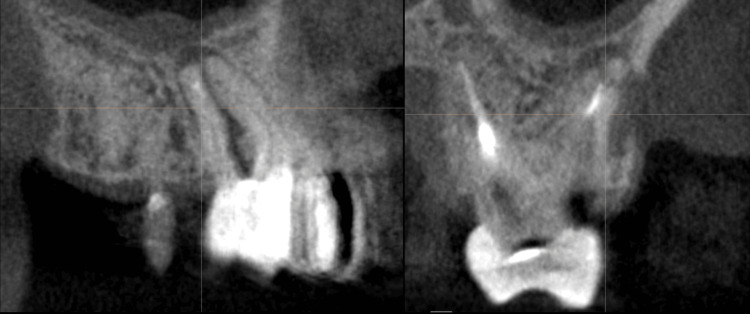
Cone-beam computed tomography (CBCT) showing extensive bone loss in the region of tooth 16 (upper right)

CBCT revealed extensive bone loss on tooth 16, predominantly affecting the buccal wall and apical aspects of the buccal roots, making its preservation unfeasible. CBCT evaluation was performed on cross-sectional slices with 1 mm thickness, reconstructed at 2 mm intervals, with a 0.15 mm voxel size (Figure 2). 

**Figure 3:**
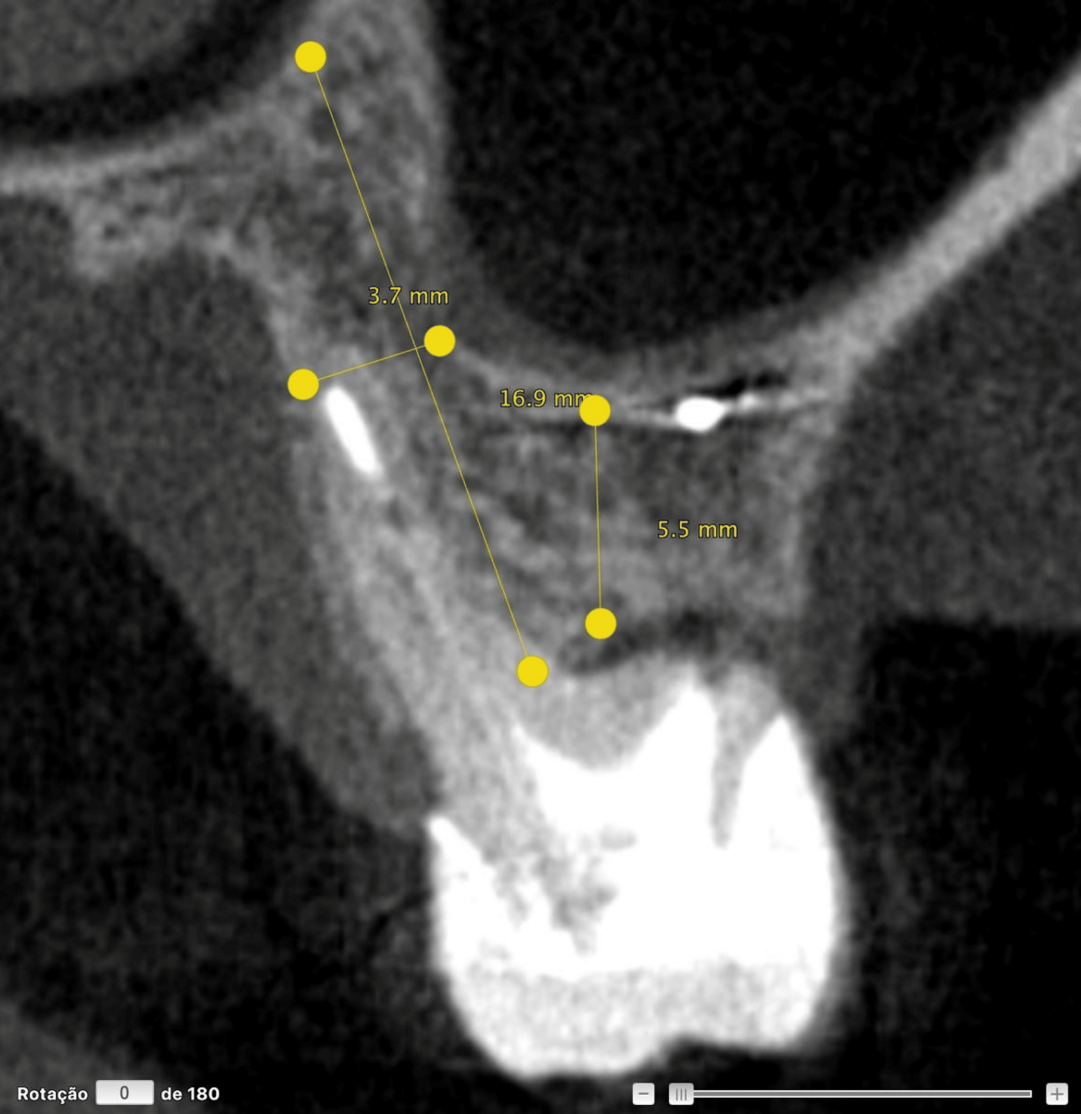
Preoperative measurements of tooth 16 in the regions of interest Residual bone height at the center of the socket, the length of the palatal bone, and the palatal-to-sinus distance (i.e., the distance between the palatal cortical plate and the maxillary sinus floor).

Several surgical prosthetic strategies have been considered for replacing tooth 16 with an osseointegrated implant, including the use of a short implant [6], direct sinus floor elevation with lateral window following extraction [6], alveolar ridge preservation with delayed implant placement [7], or even immediate implant placement within the interradicular septum, as traditionally indicated [8]; the latter two are combined with indirect sinus elevation. However, CBCT revealed maxillary sinus pneumatization extending to the furcation area, with a residual bone height limited to approximately 5 mm, precluding immediate temporization. Despite this unfavorable anatomy, the presence of a highly favorable soft tissue architecture provided the opportunity to preserve gingival contours through immediate implant placement and provisionalization (Figure 3) [9].

**Figure 4:**
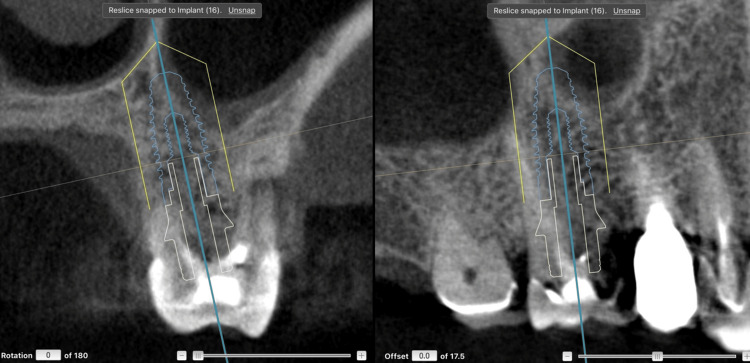
Virtual planning for implant placement in the palatal bone of tooth 16, combined with indirect sinus floor elevation, using DTX Studio Implant software Software: Nobel Biocare, Switzerland.

A thorough CBCT assessment confirmed adequate bone volume and density on the palatal aspect to accommodate an implant of sufficient length and diameter to satisfy biomechanical requirements for immediate temporization while also enabling ideal 3D positioning (Figure 4).

**Figure 5:**
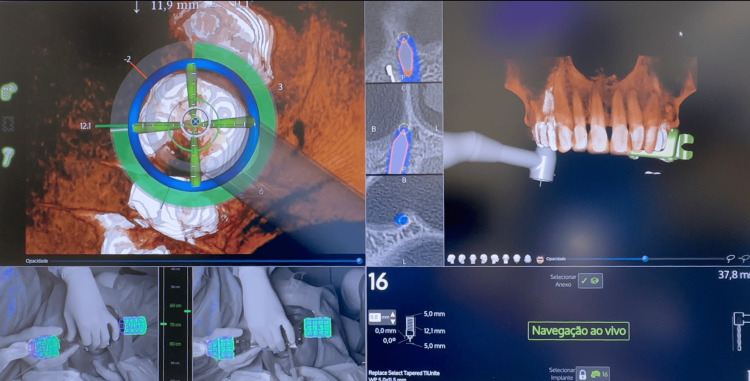
Dynamic navigation surgery with the X-Guide® system The image shows the 3D “compass” guiding the osteotomy, tomographic slices with the planned position (blue) and drill position (gray lance with orange outline), 3D reconstruction of the maxillary arch with the real-time handpiece position, camera views, and planned implant information. Dynamic navigation: X-Guide® system (X-Nav Technologies LLC, Lansdale, PA, USA).

To achieve maximum precision, DN (X-Guide®, X-Nav Technologies, LLC, Lansdale, PA, USA) surgery was performed, providing real-time guidance for drill position, angulation, and depth during osteotomy preparation (Figure 5) [4,5].

Indirect sinus floor elevation was performed using OD (Densah® burs, Versah®, Jackson, MI, USA) with VT bur sequence (VT 1525, VT 2535, and VT 3545) operated at 1100 rpm in the counterclockwise (non-cutting) direction with copious irrigation. This protocol was selected to gradually densify and expand the osteotomy while preserving bone, thereby optimizing primary stability, and it was applied according to the manufacturer’s recommendations for tapered (conical) implant osteotomy preparation to promote controlled vertical bone gain and peripheral trabecular compaction at the implant site.

**Figure 6:**
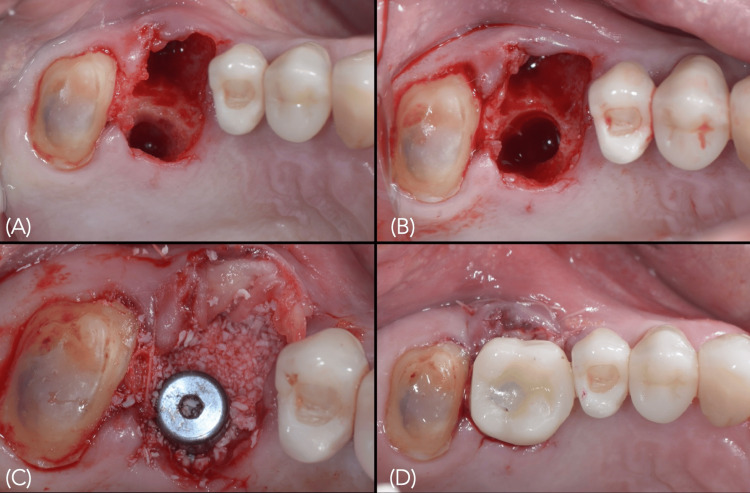
Images during implantation A) Fresh socket following extraction; B) Osteotomy preparation in the septal and palatal bone; C) Implant placement, with the gap filled using particulate xenogeneic bone substitute (Bio-Oss, Geistlich Pharma, Switzerland) and leukocyte- and platelet-rich fibrin (L-PRF) membranes sutured on the buccal aspect; D) Immediately placed implant-supported provisional restoration.

Following implant placement of 5.0 × 11.5 mm (Nobel Replace® Conical Connection, Nobel Biocare, Zurich, Switzerland), with a final insertion torque of 50 N·cm, leukocyte- and platelet-rich fibrin (L-PRF) membranes were sutured internally on the buccal aspect, and the gap between the implant and the residual socket walls was grafted with a xenograft (Bio-Oss®, Geistlich Biomaterials, Wolhusen, Switzerland). A CAD-milled provisional crown (Trilux A3 resin, VIPI, Pirassununga, SP, Brazil) was relined onto a temporary titanium abutment and maintained in infraocclusion (Figure 6).

**Figure 7:**
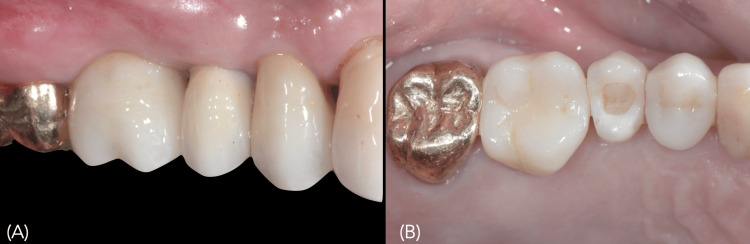
Definitive crown fabricated using the CAD-On technique and delivered to the implant A) Buccal view, and B) Occlusal view. CAD-On technique consists of the following: Zirconia abutment/zirconia framework: ICE Translucent zirconia (Zirkonzahn, Gais, Italy); Friction-fit titanium interface (Ti-interface/Ti-base): EFF Brasil (EFF Dental, São Paulo, Brazil); Lithium disilicate crown: IPS e.max CAD lithium disilicate (Ivoclar Vivadent AG, Schaan, Liechtenstein); Milling unit (CAD/CAM): CEREC MC XL (Dentsply Sirona, Charlotte, USA).

Secondary integration was inferred from clinical follow-up after the planned integration time, with no reported pain or other symptoms and no detectable mobility of the provisional crown. After four months, the case was digitally recorded using an intraoral scanner (CEREC Omnicam; Dentsply Sirona, Bensheim, Germany) to capture the maxillary arch and implant-related anatomy, and a definitive restoration was delivered, consisting of a 3 mol% Yttria-stabilized Tetragonal Zirconia Polycrystal (3Y-TZP) zirconia (Ivoclar Vivadent, Schaan, Liechtenstein) abutment with a frictional titanium interface supporting a lithium disilicate crown fused to it (Figure 7). 

**Figure 8:**
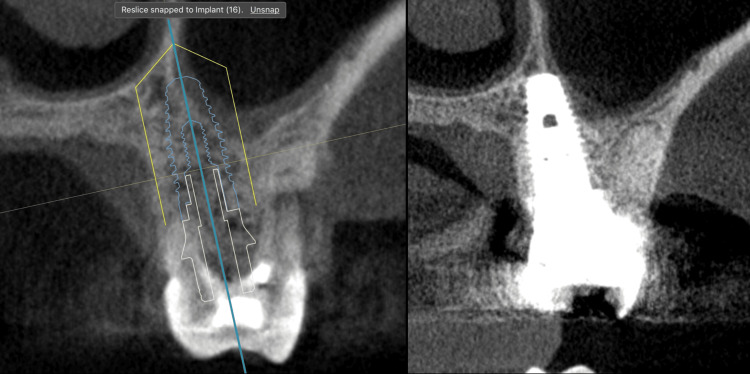
Comparison between the planned and postoperative CBCT images, showing the exact final implant position achieved with dynamic navigation.

Figure 8 presents a direct comparison between the planned (preoperative) and postoperative CBCT datasets, demonstrating that the final implant position closely matched the virtual plan achieved with dynamic navigation. 

Case 2

**Figure 9:**
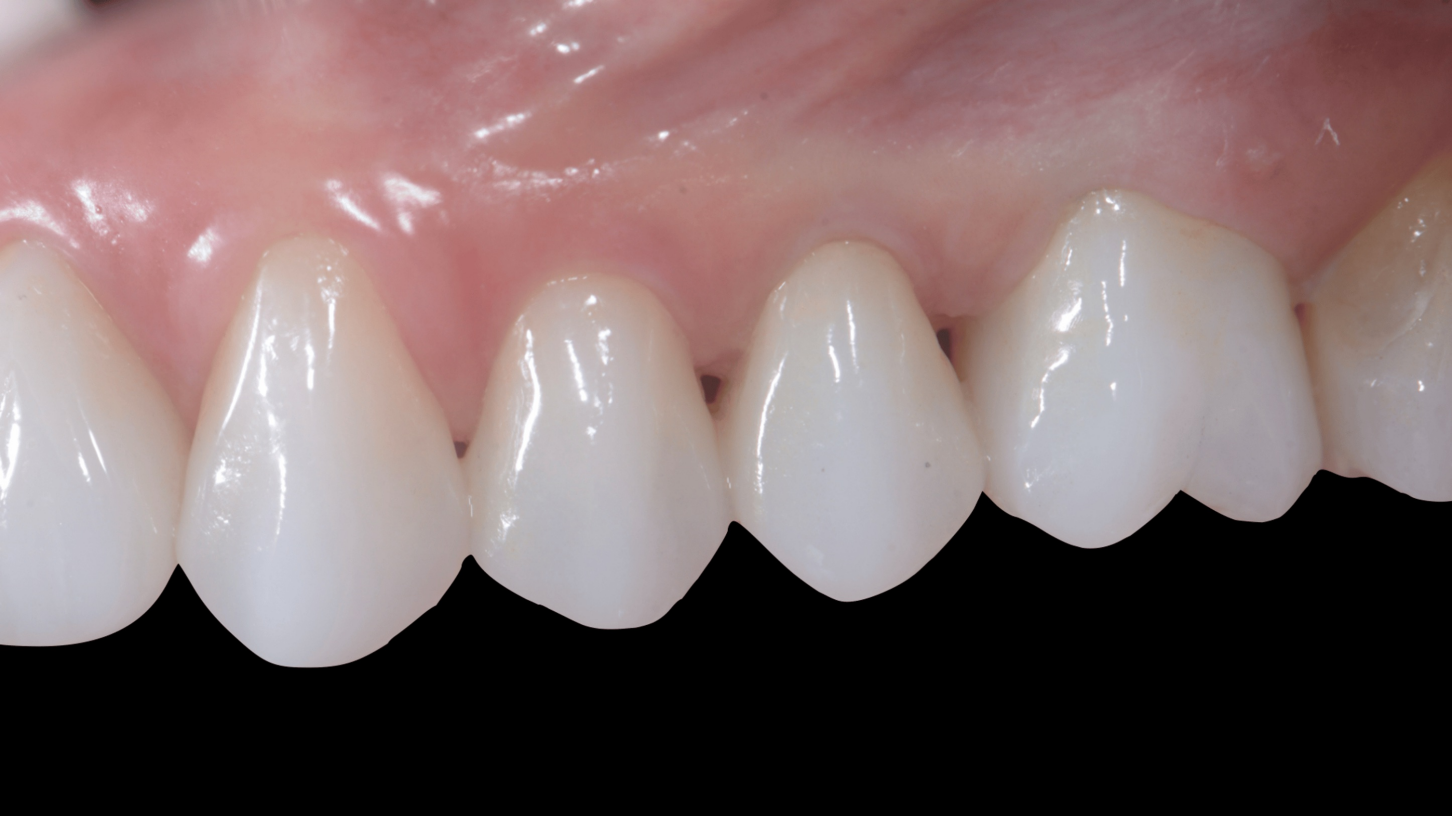
Initial presentation in case two Tooth 26 with a buccal fistula on the keratinized mucosa.

Similar to the previously described case, patient V.L.S.D. presented to our clinic with discomfort in the tooth 26 region. Clinical examination revealed a buccal fistula (Figure 9) with a 6 mm probing depth on the buccal aspect and a Class III furcation lesion between the distobuccal and palatal roots.

**Figure 10:**
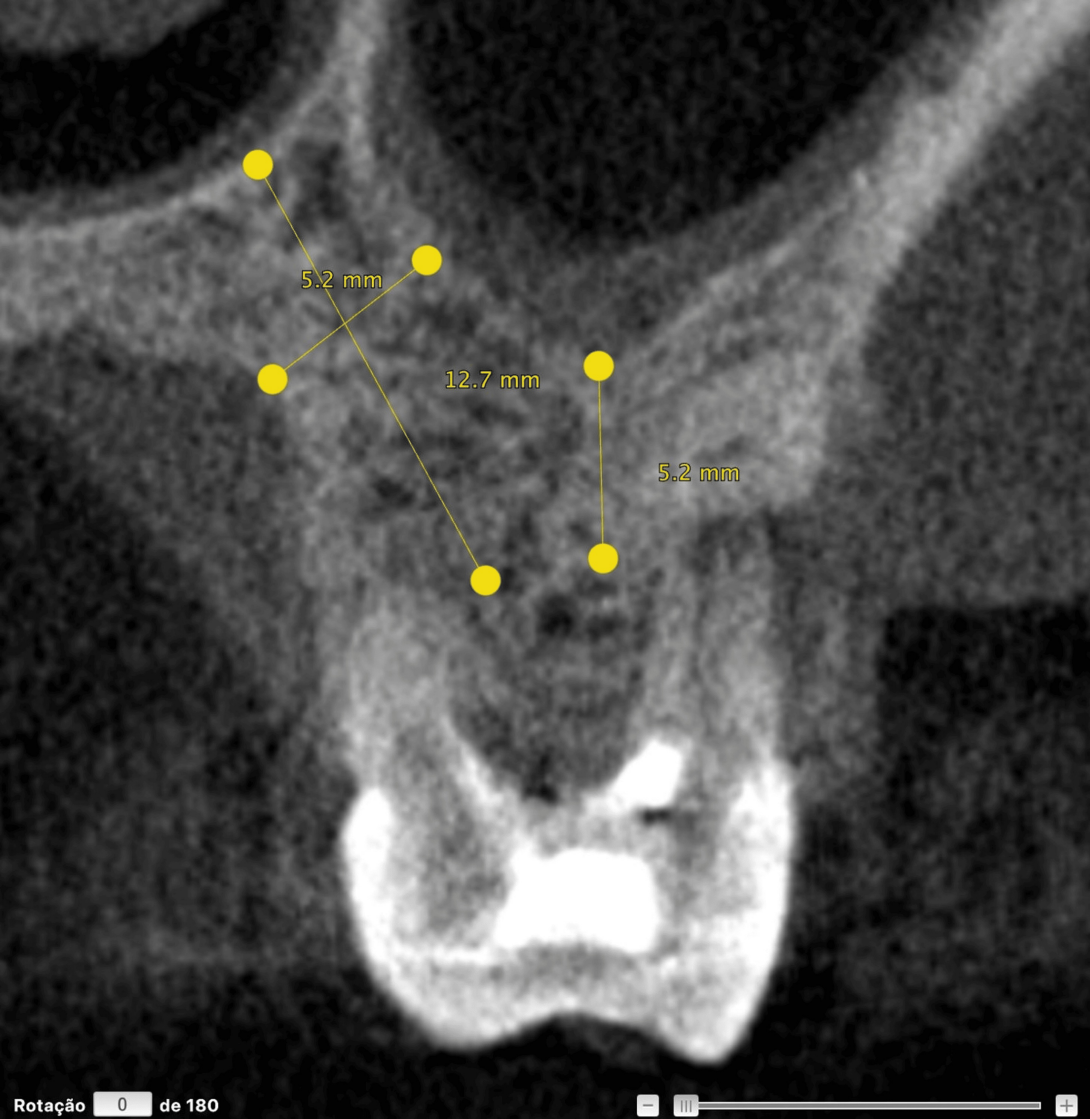
Preoperative measurements of tooth 26 in the regions of interest Residual bone height at the center of the socket, the length of the palatal bone, and the palatal-to-sinus distance (i.e., the distance between the palatal cortical plate and the maxillary sinus floor).

These findings indicate the need for extraction and replacement with osseointegrated implants. A CBCT revealed maxillary sinus pneumatization in the region of the septum of the maxillary left first molar, with a residual bone height of approximately ~5 mm (Figure 10).

Although immediate implant placement in the conventional position was technically feasible, immediate loading would have been difficult to achieve due to insufficient primary stability. Nevertheless, the soft tissue architecture remained highly favorable, reinforcing the rationale for 3D tissue preservation through immediate implant placement and provisionalization [9].

**Figure 11:**
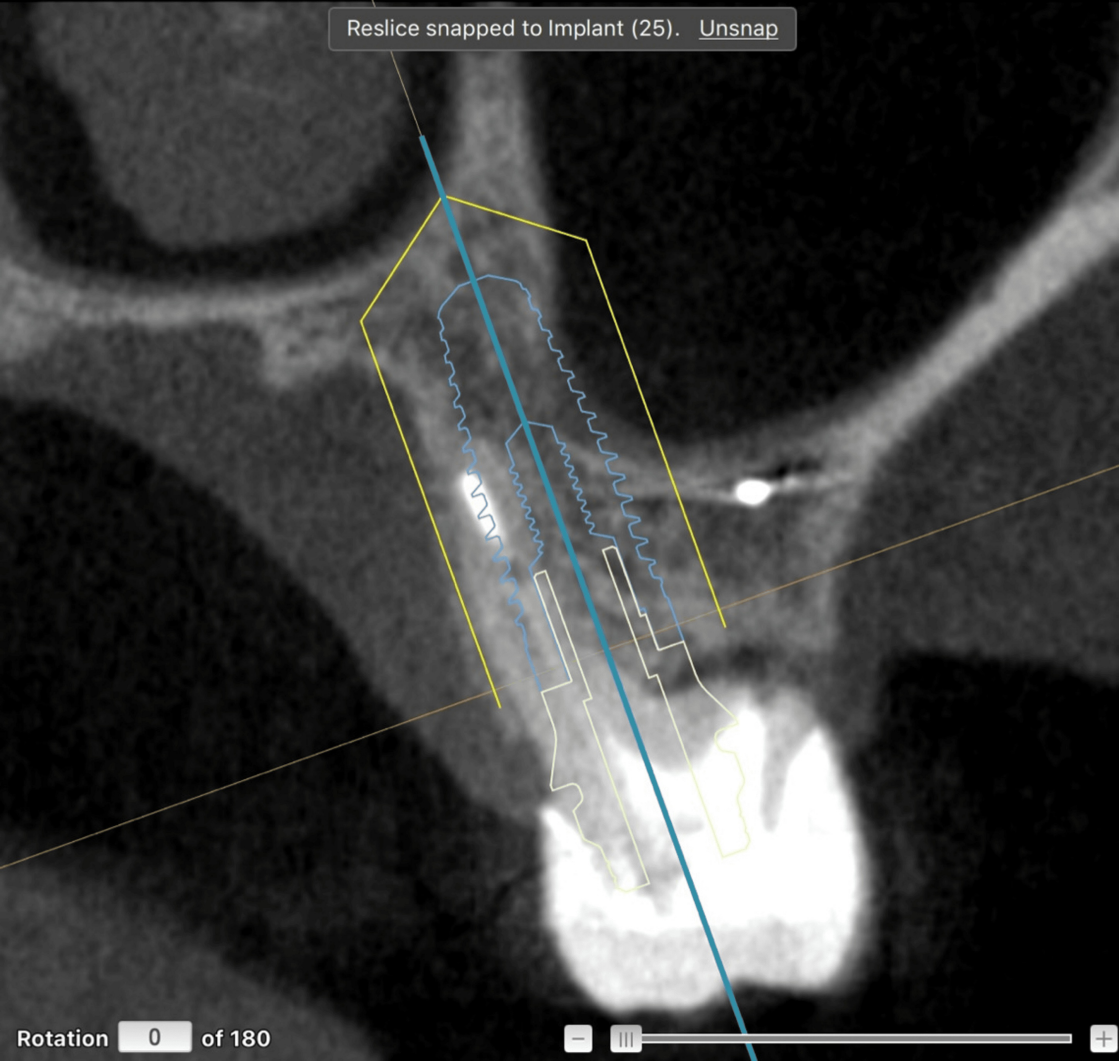
Virtual planning for implant placement in the palatal bone of tooth 26, combined with indirect sinus floor elevation, using DTX Studio Implant software Software: Nobel Biocare, Switzerland.

Another decisive factor was that tooth 25 had already been restored with a provisional implant-supported crown, presenting satisfactory clinical conditions and complete osseointegration. This allows for a splinted provisional using an osteointegrated implant, therefore enhancing the predictability of the procedure. As in the previous case, implant placement was planned in the palatal bone of site 26, where the bone volume and density were sufficient to provide adequate primary stability according to the virtual surgical plan (Figure 11).

**Figure 12:**
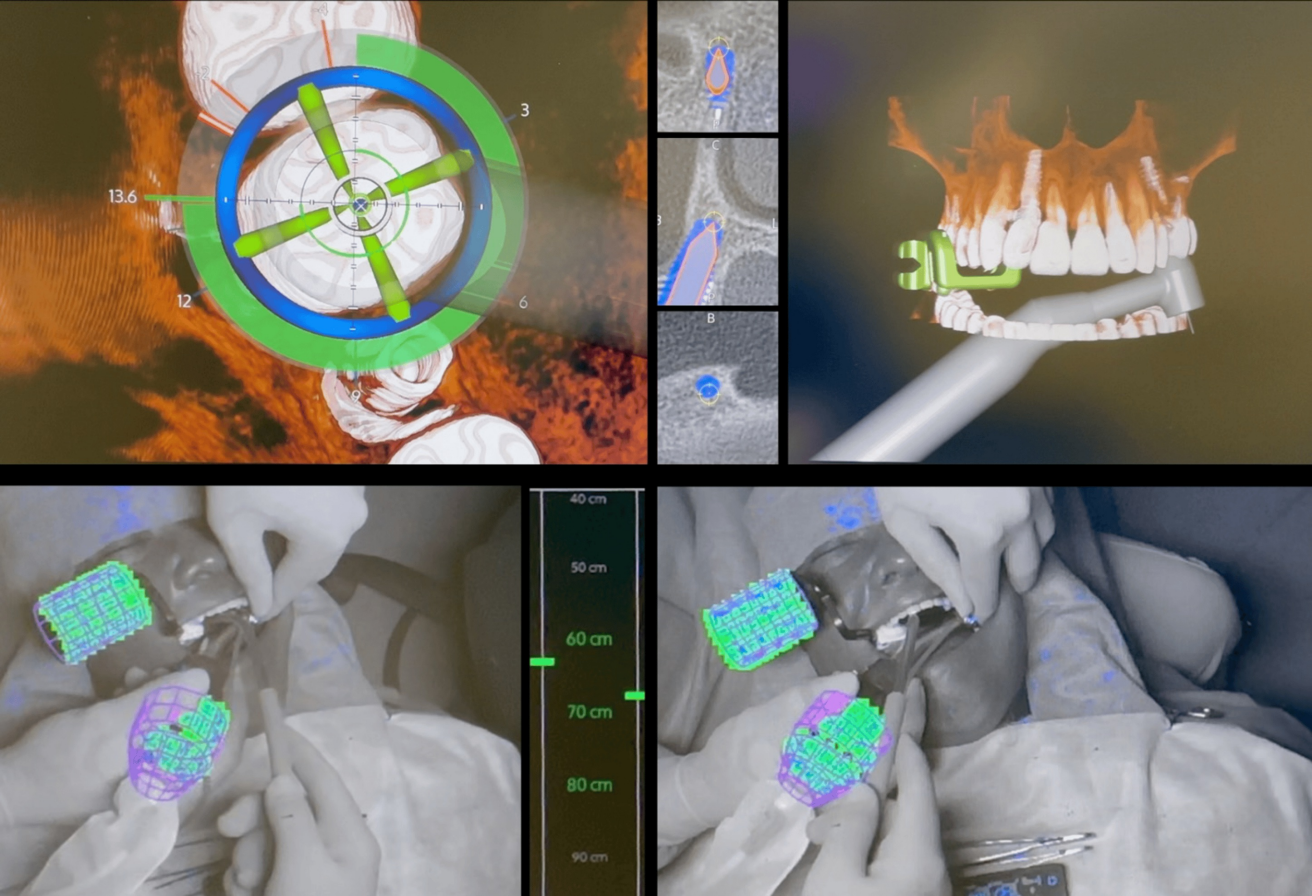
Dynamic navigation surgery with the X-Guide® system The image shows the 3D “compass” guiding the osteotomy, tomographic slices with the planned position (blue) and drill position (gray lance with orange outline), 3D reconstruction of the maxillary arch with the real-time handpiece position, camera views, and planned implant information. X-Guide® system: X-Nav Technologies, LLC, Lansdale, PA, USA.

After extraction, surgery was performed with the aid of DN in real time enabling precise control of implant angulation, depth, and overall tridimensional positioning (Figure 12).

**Figure 13:**
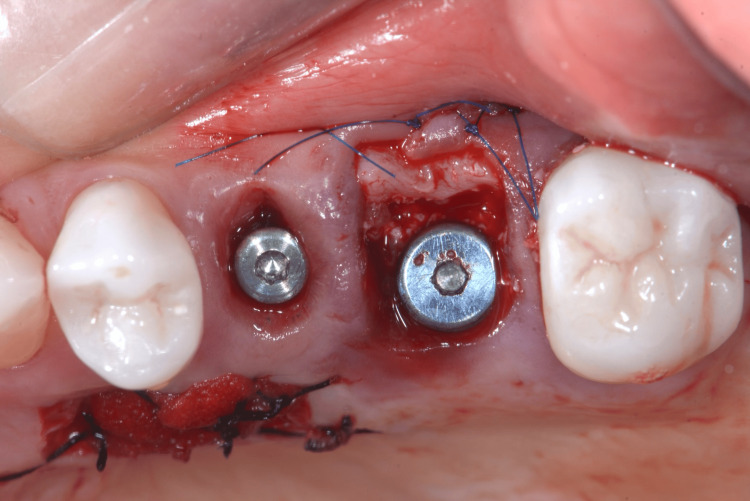
Implant placed in the fresh socket of tooth 26; the peri-implant gap was filled with a xenograft, and a connective tissue graft was sutured to the internal buccal gingival margin Xenograft (Bio-Oss, Geistlich Pharma, Switzerland).

Indirect sinus floor elevation was achieved using OD burs operated in a counterclockwise rotation (1.100 rpm) [1-3]. Following osteotomy preparation, a 5.0 × 13 mm (Nobel Replace® Conical Connection, Nobel Biocare, Zurich, Switzerland) implant was placed with a final insertion torque of 50 Ncm, peri-implant gaps were filled with particulate xenograft and a connective tissue graft harvested from the left hemi-palate was sutured to the buccal aspect to preserve the soft tissue architecture (Figure 13).

**Figure 14:**
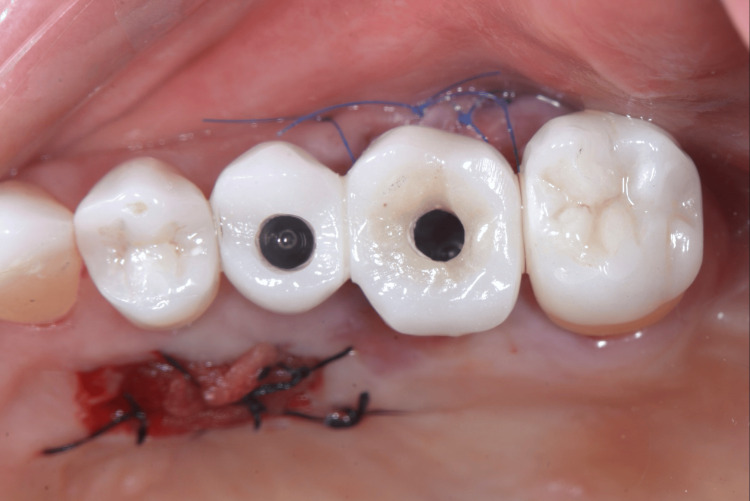
Splinted screw-retained provisional fixed prosthesis supported by the osseointegrated implant at tooth 25 and the newly placed implant at tooth 26

Immediate provisionalization was performed by splinting the provisional crowns of implants 25 and 26 (Figure 14), providing greater prosthetic stability and mechanical protection to the newly placed implant during osseointegration.

**Figure 15:**
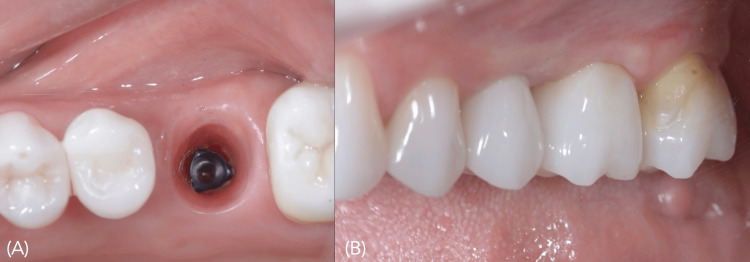
Three months after implant placement A) Healthy peri-implant emergence profile at site 26, ready for definitive ceramic restoration; B) Ceramic crowns delivered to implants at 25 and 26.

Four months after the placement of implant 26, both implants were restored using the same protocol described in the case above (Figure 15).

**Figure 16:**
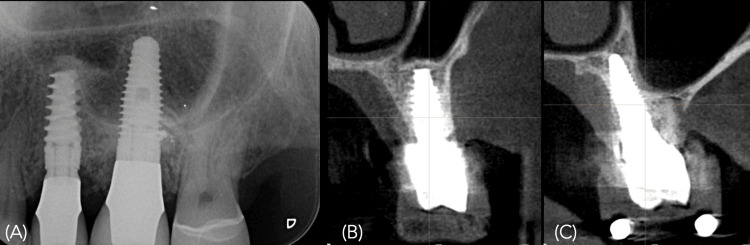
Radiographic images A) Periapical radiograph immediately after the delivery of the ceramic crowns at 25 and 26. Note that the palatal implant placement may create a radiographic impression of a lack of surrounding bone; B) Cone-beam computed tomography (CBCT) scan of the implant at 25; C) CBCT scan of the implant at 26 confirming that the final result corresponded exactly to the virtual plan, with circumferential bone surrounding the implant.

Follow-up periapical radiography and CBCT were also performed (Figure 16).

**Figure 17:**
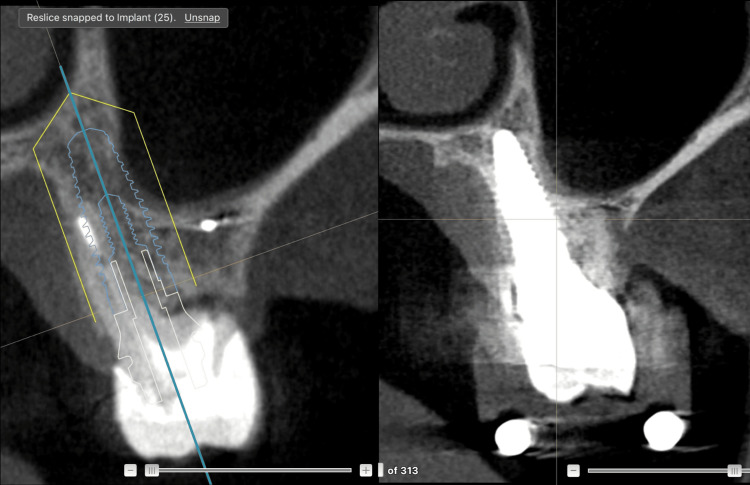
Comparison between the planned and postoperative CBCT images, showing the precise final implant position achieved with dynamic navigation CBCT: cone-beam computed tomography.

Figure 17 shows a comparison between the virtual surgical plan and the final implant position.

**Figure 18:**
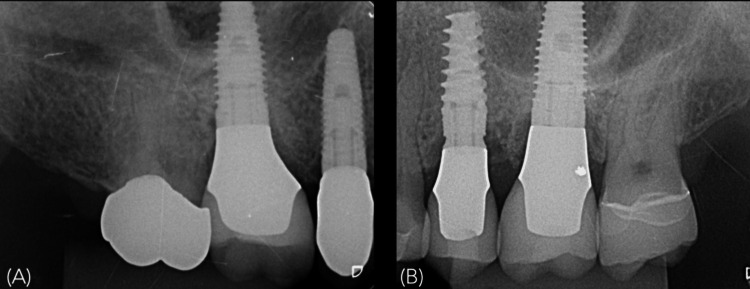
One-year follow-up radiographs Periapical radiograph obtained 12 months after implant placement in (A) Case 1 and (B) Case 2, demonstrating stable peri-implant bone levels.

Figure 18 presents the one-year follow-up periapical radiographs obtained 12 months after implant placement in both cases, demonstrating stable peri-implant bone levels. 

## Discussion

Rehabilitation with implants in the posterior maxilla is often challenging owing to low bone density and maxillary sinus pneumatization, which limit the available vertical bone height [10]. Traditionally, these situations require sinus-lifting procedures, which are frequently followed by a healing period prior to implant placement [6]. The case presented demonstrates an innovative and minimally invasive approach to overcome these challenges, combining immediate implant placement in the palatal bone, indirect sinus floor elevation with OD burs, DN surgery and immediate provisionalization. Furthermore, positioning the implant more palatally can help preserve the buccal bone plate and soft tissue architecture, a critical factor for achieving long-term esthetic and functional outcomes [9-11].

The indirect sinus floor elevation technique using OD burs is fundamental to the success of these cases. Unlike conventional drilling, this process compacts and redistributes trabecular bone laterally and apically, increasing local bone density, primary stability, and bone-implant contact while preserving the integrity of the Schneiderian membrane [1-3,12-15]. This method is less invasive than the lateral window approach and results in reduced postoperative morbidity, patient discomfort, and recovery time. Its ability to provide controlled vertical bone gain and improve the quality of the osteotomy bed significantly contributes to implant primary stability, which is an essential prerequisite for immediate provisionalization [15,16].

The DN system plays a pivotal role in enhancing the procedural accuracy and predictability, and provides real-time guidance on drill position, angulation, and depth during osteotomy and implant placement [4,5]. This is particularly advantageous in anatomically complex regions such as the posterior maxilla, where limited access and proximity to vital structures (e.g., the maxillary sinus) demand maximum precision. DN minimizes the risk of complications such as sinus membrane perforation or improper implant positioning while allowing intraoperative adjustments. Compared to freehand surgery or static guides, this approach provides greater safety, flexibility, and efficiency [17]. In a comparative study that evaluated the accuracy of three drilling systems for implants - DN, static laboratory guide, and freehand drilling - it was found that, although both guided methods outperformed the manual technique, DN presented the smallest angular and axial discrepancies. This study compared the accuracy of three drilling systems employed in dental implant placement: a navigation system, a laboratory guide, and freehand drilling. The DN and laboratory guide exhibited greater accuracy than freehand drilling. While the DN system exhibited the best performance of all three systems in terms of minimizing axial and angular errors, the DN and laboratory guide exhibited comparable performance in minimizing lateral error at entry. Although the DN is more accurate, it requires greater time and attention from both the technician and dentist for appropriate setup [18]. In a recent split-mouth study, indirect maxillary sinus elevation with immediate implant placement in the posterior maxilla was evaluated, comparing DN to the manual technique. The methodology considered outcomes of accuracy and safety, in addition to procedure time and patient satisfaction, with each participant receiving bilateral sinus elevation and immediate implants to allow for intra-individual comparisons. DN minimized intraoperative deviations and reduced the risk of complications, resulting in more predictable, efficient, and clinically favorable outcomes than freehand surgery. In complex scenarios, such as indirect sinus elevation associated with immediate implant placement, real-time navigation enhanced the 3D control of angulation, depth, and position, supporting safer workflows and greater restorative predictability [19]. Immediate implant provisionalization, performed in both cases, offers several benefits. In addition to providing an immediate esthetic and functional solution, the provisional restoration serves as a soft-tissue supporter, aiding in the preservation of the gingival architecture and emergence profile [9,18,19]. This is indispensable for optimizing the final esthetic outcomes and reducing the need for additional soft tissue conditioning. Moreover, shortening the overall treatment time and reducing the number of surgical interventions contributes to increased patient satisfaction.

The main strength of this report lies in the successful demonstration of the combined use of advanced techniques to address complex clinical challenges in the posterior maxilla. These cases illustrate how the integration of OD, DN surgery, and immediate provisionalization can result in predictable and efficient outcomes, even in anatomically unfavorable conditions. However, as this is a report of only two clinical cases, the limitations include the inability to generalize the results to a broader population. Larger studies with longer follow-up are required to validate the success and long-term success of this approach.

## Conclusions

Immediate implant placement in the posterior palatal bone combined with indirect sinus floor elevation using OD burs and DN, followed by immediate provisionalization, is a promising strategy for managing challenging posterior maxillary rehabilitation. By improving osteotomy density and primary stability while enabling real-time 3D control of angulation, depth, and positioning, this workflow may reduce surgical morbidity and enhance restorative predictability in anatomically unfavorable sites. However, the absence of objective metrics (e.g., insertion torque and/or implant Stability Quotient (ISQ)) and the limited follow-up restrict the extrapolation of these findings and do not allow definitive claims regarding protocol predictability or long-term outcomes. Therefore, further prospective studies with larger samples and longer follow-up are required to confirm reproducibility and long-term stability.

## References

[1] Mehrotra S, Varghese J (2024). Technical concepts in the management of posterior maxillary implants: a review update. Curr Oral Health Rep.

[2] Huwais S, Mazor Z, Ioannou AL, Gluckman H, Neiva R (2018). A multicenter retrospective clinical study with up-to-5-year follow-up utilizing a method that enhances bone density and allows for transcrestal sinus augmentation through compaction grafting. Int J Oral Maxillofac Implants.

[3] Huwais S, Meyer EG (2017). A novel osseous densification approach in implant osteotomy preparation to increase biomechanical primary stability, bone mineral density, and bone-to-implant contact. Int J Oral Maxillofac Implants.

[4] Aghaloo T, Hadaya D, Schoenbaum TR, Pratt L, Favagehi M (2023). Guided and navigation implant surgery: a systematic review. Int J Oral Maxillofac Implants.

[5] Block MS, Emery RW, Cullum DR, Sheikh A (2017). Implant placement is more accurate using dynamic navigation. J Oral Maxillofac Surg.

[6] Thoma DS, Zeltner M, Hüsler J, Hämmerle CH, Jung RE (2015). EAO supplement working group 4 - EAO CC 2015 short implants versus sinus lifting with longer implants to restore the posterior maxilla: a systematic review. Clin Oral Implants Res.

[7] Bassir SH, El Kholy K, Chen CY, Lee KH, Intini G (2019). Outcome of early dental implant placement versus other dental implant placement protocols: a systematic review and meta-analysis. J Periodontol.

[8] Rosa JC, Rosa AC (2025). New guidelines for the treatment of the alveolar septum in the immediate dentoalveolar restoration technique associated with osseodensification: a case series. Int J Periodontics Restorative Dent.

[9] Kan JY, Rungcharassaeng K, Deflorian M, Weinstein T, Wang HL, Testori T (2018). Immediate implant placement and provisionalization of maxillary anterior single implants. Periodontol 2000.

[10] Mattar H, Nasr TA, Keraa KM, El Sholkamy M (2025). Implant outcomes in crestal versus open sinus elevation: a randomized clinical trial. Int J Oral Maxillofac Implants.

[11] Funato A, Salama MA, Ishikawa T, Garber DA, Salama H (2007). Timing, positioning, and sequential staging in esthetic implant therapy: a four-dimensional perspective. Int J Periodontics Restorative Dent.

[12] Lima Monteiro F, Moreira CL, Galego Arias Pecorari V, Cardona Orth C, Joly JC, Peruzzo D (2024). Biomechanical and histomorphometric analysis of osseodensification drilling versus conventional technique: a systematic review and meta-analysis. Quintessence Int.

[13] Orth CC, Haas AN, Peruzzo DC, da Silva RC, de Carvalho PF, de Barros Carrilho GP, Joly JC (2022). Primary stability of dental implants installed using osseodensification or bone expansion drilling systems: a comparative clinical study. J Int Acad Periodontol.

[14] Mazor Z, Gaspar J, Silva R (2024). Maxillary sinus membrane perforation rate utilizing osseodensification-mediated transcrestal sinus floor elevation: a multicenter clinical study. Clin Implant Dent Relat Res.

[15] Gaspar J, Botelho J, Proença L, Machado V, Chambrone L, Neiva R, Mendes JJ (2024). Osseodensification versus lateral window technique for sinus floor elevation with simultaneous implant placement: a randomized clinical trial on patient-reported outcome measures. Clin Implant Dent Relat Res.

[16] Orth CC, da Silva RC, de Barros Carrilho GP, de Carvalho PF, Joly JC, Haas AN (2025). Vertical bone gain post-sinus lifting and simultaneous implant placement with osseodensification: a retrospective study. Clin Implant Dent Relat Res.

[17] Yang M, Ma Y, Han W, Qu Z (2024). The safety of maxillary sinus floor elevation and the accuracy of implant placement using dynamic navigation. PLoS One.

[18] Chen CK, Yuh DY, Huang RY, Fu E, Tsai CF, Chiang CY (2018). Accuracy of implant placement with a navigation system, a laboratory guide, and freehand drilling. Int J Oral Maxillofac Implants.

[19] Jain S, Nagy K, Bhalerao A (2025). Accuracy and safety of dynamic navigation vs. freehand approach in indirect sinus lift and immediate implant placement: a split mouth clinical study. J Dent.

